# Viral Infections and Male Infertility: A Comprehensive Review of the Role of Oxidative Stress

**DOI:** 10.3389/frph.2022.782915

**Published:** 2022-02-03

**Authors:** Roland E. Akhigbe, Sulagna Dutta, Moses A. Hamed, Ayodeji F. Ajayi, Pallav Sengupta, Gulfam Ahmad

**Affiliations:** ^1^Department of Physiology, Ladoke Akintola University of Technology, Ogbomoso, Nigeria; ^2^Reproductive Biology and Toxicology Research Laboratories, Oasis of Grace Hospital, Osogbo, Nigeria; ^3^Department of Oral Biology and Biomedical Sciences, Faculty of Dentistry, MAHSA University, Jenjarom, Malaysia; ^4^Brainwill Laboratories, Osogbo, Nigeria; ^5^Department of Physiology, Faculty of Medicine, Biosciences and Nursing, MAHSA University, Jenjarom, Malaysia; ^6^Redox Biology Group, Discipline of Pathology, Faculty of Medicine and Health, Charles Perkins Centre, The University of Sydney, Sydney, NSW, Australia

**Keywords:** antiviral, apoptosis, infertility, inflammation, oxidative stress, oxidative stress, retroviral, virus

## Abstract

Viral infections have been a part of human existence to date, though viruses have posed a huge threat with various outbreaks lately. These threats are associated with reproductive health challenges, especially male infertility. The prime focus of this review is to highlight the mechanisms associated with viral infection-induced male infertility/subfertility and identify new treatment strategies with the aim to preserve male fertility. The reviewed data showed that viral infections stimulate inflammatory responses, resulting in the release of proinflammatory cytokines, which induces oxidative stress. This oxido-inflammatory cycle could continue in a vicious cycle and threaten male fertility. Existing data from human and experimental studies show that viral infection-induced oxido-inflammatory response results in testicular damage, atrophy of the seminiferous tubules and Sertoli cells, and reduced Leydig cell mass. This is accompanied by reduced circulatory testosterone, impaired spermatogenesis, reduced sperm motility, lipid peroxidation, DNA fragmentation and apoptosis of the sperm cells. Based on the available pieces of evidence, antioxidant therapy, *in vivo* and *in vitro*, may be beneficial and protects against the potential risk of male infertility from viral infection. It is, however recommended that more clinical studies be conducted to demonstrate the possible protective roles of antioxidants used as adjuvant therapy in viral infections, and in the *in vitro* treatment of semen samples for those utilizing semen washing and artificial reproductive techniques.

## Introduction

Infectious diseases have been inflicting humans from time immemorial. This has worsened in the last four decades with various viral outbreaks. The most mention-worthy viruses include the Human Immunodeficiency Virus (HIV) that emerged in 1981, Severe Acute Respiratory Syndrome Coronavirus (SARS-CoV) that emerged in 2002, outbreaks of influenza A virus subtype H1N1 (A/H1N1) H1N1 strains (1918 flu pandemic, the 1977 Russian flu pandemic as well as the 2009 swine flu pandemic), Middle East Respiratory Syndrome Coronavirus (MERS-CoV) (caused epidemic in 2012), and Ebola virus (outbreak in 2013) and the Severe Acute Respiratory Syndrome Coronavirus-2 (SARS-CoV-2) causing Coronavirus disease-2019 (COVID 19) that emerged in 2019 and is still on-going (1–3). The emergence of infectious diseases has a direct influence on human health outcomes, ultimately impacting sustainable development (4). By the end of the twentieth century, an estimated 34.3 million individuals globally were infected with HIV/AIDS (5). Millions of children were orphaned due to the epidemics, which also undermined social life and corroded civic order and economic progress (6). The SARS-CoV pandemic had deadly consequences, affecting 8,098 persons, resulting in 774 fatalities by February 2003 (7). However, in many of the afflicted areas, the outbreak revealed some flaws in hospitals and community control mechanisms (8). The 2009 H1N1 influenza pandemic had a global impact, affecting 214 nations and resulting in 18,449 fatalities. With earlier influenza epidemics posing a persistent threat, scientific communities were adequately equipped in terms of frame of mind and infrastructure, allowing for faster and more thorough researches on the basic features of the disease, as well as implications for its regulations and learnings to deal with future epidemics (9). MERS-CoV, causing another coronavirus epidemic, has one of the highest case fatality rates among previous pandemics (43% in 147 individuals) (10). More recently, between 2013 and 2016, the Ebola virus posed a major public health threat in the afflicted nations, with significant social and economic consequences (11). As the new decade begins, the world is struggling to contain another pandemic-strength virus, the SARS-CoV-2 causing COVID 19. It is one of the most serious public health crises in human history. The virus was originally discovered in December 2019 and isolated from a group of pneumonia-stricken employees at the Wuhan seafood market in China (12–14). Shortly later, on March 11, 2020, the World Health Organization (WHO) proclaimed it a worldwide pandemic (15).

Viral infections lead to a multitude of health issues which mandates understanding the impact of these viruses on the vital physiological processes. Reports on the association of viral infection on female reproductive functions are profound, but the mechanism by which these viruses affect male fertility still awaits in-depth explanation. Infection is considered to be a contributing factor in male infertility. Certain infections, such as genital tract infections, have been proven to have a detrimental impact on sperm quality (16). Microbial infections and the subsequent inflammatory responses may disrupt the endogenous environment of the male reproductive system resulting in inflammation of the male reproductive organs. Reproductive tract infections-induced immunogenic dysregulations are responsible for about 15% of male infertility cases (17, 18). Activating the innate immune system, which typically disturbs the testis-specific restrictive immunological responses and leads to leukocyte infiltration and elevation of proinflammatory mediators, is a testicular strategy for fighting viral infections (19, 20). The inflammatory reactions may cause oxidative stress (OS), which is considered to be the central mechanism that elicits oxidative damage in the male reproductive tissues, affects every testicular cell and also lead to the disintegration of the physical barriers of the testes (17–23). These intricate processes of testicular responses to viral infections are still evolving. Thus, the present review aims to provide a comprehensive understanding of the mechanisms by which viral infections affect male reproduction and how intricately OS mediates the infection-induced male reproductive tissue disruptions.

## Influenza Virus and Male Fertility

Direct viral invasion or secondary inflammatory pathways are two ways viral infections can influence both male and female reproductive processes. The effects of H1N1 on the quality of human sperm have been confirmed by a large body of evidence (24, 25). The flu can have long-lasting effects on sperm DNA integrity, and it can cause the transitory discharge of defective sperm. It has even been linked to an increased risk of infertility in some studies (24, 26).

Sergerie et al. (26) revealed that a febrile episode could negatively impact on sperm parameters and DNA integrity. Evenson et al. (24) investigated the characteristics of human sperm chromatin structure following a bout of influenza and a high fever and discovered that influenza might have latent effects on sperm chromatin structure, resulting in the transitory release of aberrant sperm. Several studies using animal models demonstrate that influenza can damage sperm quality and result in infertility (25). Devi et al. (27) showed in a mouse model that the human influenza virus can cause spermatozoa to develop chromosomal aberrations. While data suggests that a local inflammatory reaction is unlikely, the temporary sperm abnormalities seen are most likely the result of a combination of systemic fever and direct DNA damage, resulting in apoptosis and a sharp decline in fertility (28, 29).

As a result, solid evidence supporting the influence of influenza infection on sperm quality may be drawn. It is fascinating to consider whether influenza can cause future infertility. On this subject, data are scarce. Evenson et al. (24) propose that influenza has a latent effect on sperm, which could result in infertility. To sum up, more research on the link between influenza and infertility is still needed.

## Zika Virus and Male Fertility

Studies have revealed that ZIKV thrives in the male genital tract. The virus is expressed in the semen of symptomatic Zika-infected men (30), and the ZIKV RNA has been reported to persist longer with a higher concentration in the semen than in the blood (31). Mansuy et al. (32) reported that the ZIKV RNA could be detected in the semen till the 188th day following infection. Also, it has been revealed that the clearance time is 25–83 days in the semen, while it is 5–15 days in the circulation (30, 33–35). Hence, it could be inferred that the male urogenital tract serves as a reservoir for this virus. The testes seem to be the organ of choice for ZIKV replication because they are capable of sustaining high viral load for a prolonged period (36–42). Although most of the ZIKV RNA in the semen does not have an infectious virus, implying that they loss their infectivity; the presence of the virus in the male urogenital tract remains a threat to male fertility as the length of days the virus will retain its infectivity in the male urogenital tract is yet unclear.

ZIKV can cause orchitis, epididymo-orchitis, and testicular atrophy, leading to infertility and hypogonadism in experimental animals (40, 43). Although the circulatory level of testosterone is unaltered during the acute phase infection (44), ZIKV decreases sperm count and increases abnormalities in sperm morphology up to 90 days post-infection (44). In a prospective follow-up study in Brazil, Avelino-Silva et al. (45) demonstrated that ZIKV infection led to a long-term detrimental effect on human male fertility evident by abnormal spermogram at 12 months post-infection. An earlier study by Govero et al. (43) in mouse-model showed that the initial ZIKV infection-induced damage was observed 2 weeks after infection, with germ cell loss and impairment of Sertoli cell function evident by reduced concentrations of circulatory inhibin B. With persistent infection, most germ cells were lost, and spermatogenesis was almost utterly lost on day 21. Govero et al. (43) also demonstrated significant expression of ZIKV in epididymal sperm cells seven days post-infection. This suggests that ZIKV may distort spermatogenesis and also cause direct damage to the spermatozoa and testis (46). The role of the Sertoli cells in maintaining immune privilege has been proposed to be beyond a simple barrier function but possibly dependent on secreted and surface immunosuppressive and immunoregulatory factors (47). During infection, these protective factors that maintain an anti-inflammatory immune environment in a physiological state may render the germ cells susceptible to microbial/viral attack.

## Mumps Virus and Male Fertility

The hallmark of Mumps infection is bilateral swelling of the parotid gland (parotitis). Other than parotitis, inflammation of the testis (orchitis), usually unilateral, is the most common manifestation of Mumps, and affects 10–20% of adult men (48). This can negatively impact male fertility. Testicular swelling and tenderness, and scrotal inflammation are typical of Mumps orchitis. In many cases, inflammation of the epididymis (epididymitis), particularly the head region, is associated with orchitis, a condition usually termed epididymo-orchitis (49, 50). This implies that the infection does not only negatively impact testicular function, but also sperm function, when the viruses are released into the epididymis. The acute orchitis can resolve in 2 weeks but can significantly impair testicular function, including testicular atrophy reported in nearly half of the cases. Sperm count, motility and morphology have been shown to remain drastically affected even years after recovery, indicating that the virus impacts testicular and sperm functions (48).

Humans are the only natural reservoir of Mumps virus replication, and studies on viral pathogenesis are scarce. Differential response of testicular cells to viral replication is reported. For example, a mouse study showed that the virus can infect Leydig cells, Sertoli cells, germ cells and resident testicular macrophages (51). However, the viral replication was higher in Sertoli cells compared to Leydig cells and testicular macrophages, whereas germ cells were resistant to replication, indicating that testicular cells have a variable innate response to Mumps virus infection (51).

The acute inflammation in orchitis can lead to the release of inflammatory cytokines, which can impair organ function through oxidative stress-mediated pathways, resulting in disruption of testicular function. Increased levels of TNF-α were associated with Mumps and concomitant reduction in the testosterone synthesis by Leydig cells (52), possibly through inhibiting the functions of enzymes involved in testosterone synthesis. Further, pathological levels of TNF-α can disrupt the blood-testes-barrier (BTB) by impacting the levels of junctional complexes occludens and zonula occludens 1 as the primary target of TNF-α (53). The development of autoantibodies against GCs during orchitis is common and could result from disrupted BTB leading to infertility in young adults where spermatogenesis is active compared to children when it is inactive (54).

In summary, Mumps can have a drastic impact that may involve germ cell apoptosis, reduced sperm count, motility, morphology and testicular atrophy, all leading to male infertility. Therefore, vaccination is the solution to these infections. Further, due to limited data on pathogenesis studies of Mumps associated orchitis, experimental animal studies are critical.

## HIV and Male Fertility

The virus has two subtypes; HIV-1 and HIV-2, which are primarily transmitted *via* sexual intercourse, vertical transmission, sharing of sharps and blood and blood products (55–57). HIV-1 is expressed in the semen of asymptomatic HIV-infected men as free HIV-1 RNA particles in the seminal plasma and as a cell-associated virus in non-spermatozoal cells like the lymphocytes and macrophages (58). Most HIV-1 RNA likely originates from the seminal vesicles and prostate since the concentration of HIV-1 RNA in the semen is not altered by vasectomy (59). Intermittent shedding of HIV-1 RNA is the commonest pattern of HIV-1 expressed in semen (60, 61). This is partly due to the varying composition of the ejaculate between men, and overtime in the same individual. The rise in HIV-1 RNA levels in semen independent of the circulatory levels of HIV-1 RNA levels following local inflammation also explains this phenomenon. Intermittent shedding may lead to discrepancies between circulatory and seminal plasma concentrations of HIV-1 RNA.

Although in rare cases, HIV-infected patients have been reported to have high semen viral load, usually, HIV load is lower in the seminal fluid than in the blood (62, 63). HIV has the propensity to integrate its DNA into the host genome. Studies have shown that HIV leads to chronic orchitis, resulting in hypergonadotropic hypogonadism with impaired steroidogenesis (64). It has also been revealed to cause oligozoospermia and teratozoospermia, usually secondary to HIV-induced hypogonadism and leukocytospermia (65). In addition, HIV triggers Sertoli-cell-only syndrome, germ cell damage and peritubular fibrosis. Studies have demonstrated that HIV reduces sperm motility and the percentage of spermatozoa with normal morphology (66, 67). Similarly, van Leeuwen et al. (68) reported a decrease in semen volume and sperm motility in multiple semen samples obtained before and after seroconversion for HIV-1 from a single donor. AIDS patients have been reported to have low circulatory testosterone and high levels of LH and FSH. The reduced testosterone level may be due to the fibrosis of the testicular interstitial tissue and reduced Leydig cell number (69). The HIV-associated rise in circulatory cytokines have also been reported to contribute to depressed testosterone synthesis (70).

Despite the effectiveness of highly active antiretroviral therapy (HAART) in the management of HIV/AIDS, it has also been implicated in infertility. Akhigbe et al. (71) demonstrated that HAART impaired male sexual acts *via* a testosterone-dependent mechanism and hyperprolactinemia. Administration of HAART has also been revealed to induce extensive atrophy of the seminiferous tubules and hypocellularity. Furthermore, HAART impairs spermatogenesis and steroidogenesis, resulting in reduced serum testosterone, semen volume and sperm motility (72, 73). In an attempt to evaluate the associated mechanism of HAART-induced male reproductive toxicity using a rat model, Akhigbe et al. (74) observed that HAART led to lipid peroxidation and inflammation of the testis. This was associated with reduced testicular and sperm DNA integrity and dysregulation of lactate transport and glutathione content.

## Herpes Simplex Virus and Male Fertility

The transmission of Human Papilloma virus (HPV), for the most part, is by way of the sexual itinerary (75). The genital passage is infected by a relative amount of 30–40 varieties of HPV from the α-genus (76, 77). It is supposed that a greater part of men who engage in sexual activity tend to obtain genital HPV infection during their lifetime, although around 90% of these infections are not clinically significant, and a better part of the infection tend to resolve impulsively (78). HSV-1 and HSV-2 can be transmitted sexually. HSV, especially HSV-2, has been detected in the semen (79) and could likely be internalized into the sperm cells. HSV infects the penile mucosa and triggers a chronic infection (80). The virus replicates in the keratinocytes and then spreads to the nerve cells, where it rests in the latency phase. HSV returns to the epithelial surface upon reactivation, inducing cell death and vascular lesions (81). Although the seminiferous tubules seem to be spared due to the protection conferred by the blood-testicular barrier, HSV-2 infect almost all tissues/organs of the male genital tract (82, 83). The prevalence of HSV shedding in seminal fluid ranges between 0 and 100% in chronic infection (84–88).

Findings reporting the presence of HSV DNA in oligozoospermic samples suggest that HSV may cause infertility/subfertility. Although studies confirming HSV-induced infertility are rare, HSV has been associated with low sperm count and reduced sperm motility (89, 90). The virus's ability to infect almost all organs of the male reproductive tract could infer that it has the propensity to cause direct damage to the spermatozoa and alter the sperm quality (such as reducing sperm count, motility and sperm cells with normal morphology). During ART, HSV can be transmitted vertically through the sperm cells, thus increasing the risk of miscarriage and adverse effects to the fetus (91).

## Human Papillomavirus and Male Fertility

The infection of seminal fluid is of great medical importance in the etiology of male infertility, which in most cases is not unconnected with defective semen quality (92, 93). Several studies have documented the transmission of HPV through sexual intercourse in males. It has been reported that about 16% of semen from men seeking fertility assessment and, or medical care had HPV in their semen and a 10% prevalence among the population as a whole (94).

In addition, HPV infection has been implicated as a worthy risk factor for infertility in men (95, 96). Though the functionality of the process that implicates HPV seminal infection with male infertility is shrouded in uncertainty (95), it has been proven that HPV infection considerably causes a reduction in essential sperm indices (96). A good indicator of sperm motility that has been generally accepted is sperm progressive motility which is perceived as a basic functional parameter needed for fertilization (97). A good number of researches has shown that HPV infection is implicated in male infertility with a reduction in the progressive motility of the sperm (98–106). On the contrary, Zheng et al. (107) established that there was no clear dissimilarity of sperm progressive motility proportion amidst infected and non-infected infertile males. The systematic review and meta-analysis explored the likely impingement of HPV infection in semen on the progressive motility of sperm cells in infertile males; however, the evidence is far from conclusive because of the sample sizes and existing confounding factors of the currently available studies.

## Hepatitis Virus and Male Fertility

The prevalence of viral shedding in the seminal fluid is about 68% in chronic infections (108). Similarly, the prevalence of HCV in seminal fluid varies from 29 to 39% in the early-stage to 32–46% in chronic infections (109–113). Some studies documented the presence of HBV-DNA in the semen 120 days after it was no longer seen in the serum (114). Huang et al. (115) observed that HBV-infected patients have higher sperm chromosomal aberrations than the controls. Transfection of sperm cells with HBV has been revealed to induce apoptosis and decline fertilization capacity (116). Data on the impact of HCV on semen quality is conflicting. A study reported that HCV-infected semen does not alter semen quality, importing that HCV is likely not to exert a negative impact on sperm cells (117). Interestingly, other studies demonstrated that HCV reduced sperm count and motility and increased abnormal sperm morphology (118, 119). In addition, HCV has been reported to reduce circulatory testosterone and inhibin B (118–123). A recent study revealed that oxidative stress is implicated in the pathogenesis of HBV-induced sperm damage. HBV enhances reactive oxygen species (ROS) generation, resulting in oxidative damage of the spermatozoa and increased sperm DNA fragmentation and chromosome mutations (124).

## Parvovirus and Male Fertility

The discovery of AAV vector DNA in the semen of patients enrolled in a gene therapy trial for hemophilia B based on the intramuscular delivery of AAV vectors carrying the factor IX gene suggested for the first time that semen infection could occur after delivery of engineered AAV vectors for gene therapy (125). This raised concerns on the danger of germline transduction and germline transmission risk associated with AAV vectors were investigated in different male laboratory animal models (126). Indeed, the seminal dissemination of the vector was greater with intravascular delivery than when it was injected intramuscularly. The sequences of the AAV vector were transiently detected, and the detection depended on the dose and time (127–129). With extensive follow-up and observation of numerous spermatogenic cycles, it was suggested that these vectors did not appear in recurrence in rabbit semen and thus bear low possibility of germline transmission for humans as well (127–129). It was also reported that in vasectomized rabbits, even in the absence of germ cells, the viral vector could contaminate the semen (128). This may indicate that the viral component may be transmitted *via* the seminal plasma alone, irrespective of the sperm concentration. Moreover, the viral vectors transduction directly into isolated murine spermatogonia or mature sperm could be done successfully (127), while transduction of the same in murine male germline stem cells was successful (130). Another study demonstrated that following either intraperitoneal AAV vector administration to mouse neonate or intravascular administration into mouse fetus, the viral components were persistently found in those animal gonads for over a year of follow-up. At the same time, viral components were absent in the isolated sperm DNA of these mice (131). Taken together, these findings suggest a low risk of accidental germline transmission of AAV, but precautions are needed when these viral vectors are employed for gene delivery (126). Nevertheless, in the semen of infertile men, the AAV DNA have been detected more frequent than in normal semen samples (20–40 per cent vs. 0–5 per cent, respectively) (132–134). Studies showed an association of AAV infection with oligoasthenozoospermia and oligospermia but found that the presence of AAV DNA in semen was not significantly related to semen quality, including the functional capacity of sperm (132–134).

The suggested significances of parvovirus infection in the male reproductive tract or semen include sexual transmission of infection; subfertility or infertility owing to dysfunctions of the testicular cells; viral genome incorporation into the germ cell genome and thereby transmission to female partners and infection of ova and subsequently of the embryo, which may cause miscarriage, embryonic abnormalities and also a risk of infection transmission with assisted reproduction (135). These data show that AAV infection of the male reproductive tract occurs rather often. The presence of AAV in ejaculates supports the theory that the virus is transmitted sexually, and the preference for viral DNA identification in aberrant semen samples from infertile men suggests that AAV may play a role in male infertility (135). Furthermore, as AAV DNA was found in testis biopsies in 26% of infertile (azoospermic) men (132), it is tempting to hypothesize that AAV might disrupt sperm cell development, resulting in infertile spermatozoa.

The mechanism associated with the entry of parvoviruses into the male reproductive tract, susceptibility of the testicular cells and the impact of the virus on reproductive endocrine regulations is an essential area subject to future in-depth research. In this aspect, knowledge of whether parvovirus infection affects male reproductive tissues *via* an OS-mediated pathway will help to understand the underlying pathogenesis better. The viral kinetics also needs to be elucidated as to know how the virus replicates in the male reproductive tract and whether the testis serves as a safe hide-out reservoir for the virus, as well as the testicular antiviral defense mechanisms against this virus.

## Ebola Virus and Male Fertility

Semen samples collected from recovering survivors of the Ebola virus revealed its presence in the semen. In Sierra Leone, a study conducted on 93 survivors showed that semen specimens of all men (*n* = 9) were positive for virus 2–3 months after the disease onset although the virus was undetectable in circulating blood. Likewise, 26 of 40 semen samples and 11 of 43 were positive when tested after 4–6 and 7–9 months, respectively, post-disease onset (136). These findings show that even virus is undetected in peripheral blood smears, it stays in semen for months and can be a potential source of transmission to sexual partners. For example, a female acquired the Ebola virus through sexual transition from her partner whose semen tested positive for the virus even after 199 days after the disease onset (137). The Ebola virus has been detected in the infected man even >900 days post-infection, which triggers an alarm on its long-term sexual transmission (138). These results were echoed in another study on 277 survivors where 93% of men semen specimens were positive after 3 months, and the virus was still detectable in the semen of 0.06% of the cases after 2 years of the onset of infection (139).

The virus has been detected in the testicular interstitium, seminal vesicles, and prostate thus can have direct consequences not only on the testes but accessory sex glands (140). Like the Mumps virus, the Ebola virus was also shown to be associated more with Sertoli cells and BTS disruption, which has dire consequences on endocrine and spermatogenesis functions of the testes (141). Survivors of Ebola virus infection have reported complaints of erectile dysfunction (5–8%) and reduced sexual desire (10–12%), probably due to compromised endocrine function of the testes (142, 143). However, the causal relation of reproductive dysfunction to viral infections remains elusive. Nonetheless, significant production of a suite of inflammatory cytokines, including TNF-α, has been reported in response to Ebola infection. This points toward the role of inflammation-induced oxidative stress on cell and organ functions (144).

Taken together, the available literature indicates that the Ebola virus has a strong potential for sexual transmission for several months, and the virus localizes in Sertoli cells and other parts of the reproductive tract and can impact testicular function. The high mortality of this disease and scarce data on sperm parameters is a limitation and warrants further longitudinal studies on laboratory animals, including rodents and non-human primates.

## Coronavirus and Male Fertility

Since the SARS-CoV epidemic in 2002, researchers have been reporting the possibility for coronaviruses to infect the human reproductive system (145, 146). Although studies are ongoing to understand the pathogenesis of this novel viral infection better, it has been established to be transmitted through inhalation of infected droplets and contact with infected persons, whether they are symptomatic or not (14). Coronavirus enters the human cells through endocytosis and translocation by binding to angiotensin-converting enzyme 2 (ACE 2), which primarily counterbalances ACE (147, 148). In the cell, it replicates, increases the pH of endosomes and lysosomes and activates p38 mitogen-activated protein kinases (MAPK) and extracellular-regulated protein kinases (ERK), which triggers a hyper-inflammatory response (149, 150).

When it comes to affecting reproductive functions, it has been hypothesized that SARS-CoV-2 may also activate MAPK/ERK signaling. Men have a larger expression of the ACE2 receptor than women do, which may explain why male reproductive functions appear to be farther vulnerable to the effects of SARS-CoV infection (146). It is known that sperm cells, Leydig cells, and Sertoli cells have significant levels of ACE2 receptor expression (150). However, according to certain investigations, the SARS-CoV virus and SARS-CoV-2 virus was not found in the semen samples of infected men (151, 152). The fact that SARS-CoV-2 virus particles were found in semen (153) raises questions about the validity of this claim. When regional or systemic inflammation occurs, the virus may succeed to get access to the germ cells owing to the compromised blood-testis barrier (153, 154). It is reported that serum luteinizing hormone (LH) in COVID-19 patients remain greater than in healthy men (150). On the contrary, these patients demonstrate significantly lower serum testosterone (150), and a reduced testosterone to LH and follicle-stimulating hormone (FSH) to LH ratio. These indicate that SARS-CoV-2 may have a direct impact upon testicular tissue rather than affecting the hypothalamus-pituitary-gonadal (HPG) axis (155), possibly *via* oxidative-sensitive MAPK/ERK-mediated hyper-inflammatory response.

Numerous other indirect impacts, for example, germ cell destruction and testicular dysfunction, resulting from a prolonged increase in body temperature consequent to the viral infection, have also been described. In some situations, hyperplasia of Leydig cells has been reported in patients who have been infected with the virus (153, 156). Some of the most noticeable symptoms of possible inflammatory responses resulting from SARS-CoV infection include disruption of functions of Leydig cell, thereby lower testosterone synthesis, and damage to the seminiferous epithelium causing disintegrated blood-testis barrier (146).

Studies have revealed that coronaviruses involved with previous epidemics are connected with orchitis, among which SARS-CoV is a prominent instance. This may result in the interruption of spermatogenesis and germ cell death, consequently reducing the quality of sperm produced (146). In testicular tissues, immunohistochemistry has verified the presence of IgG deposition; nevertheless, no viral genomic components were seen in either the testicular tissue or the seminal plasma. Inflammatory responses may have significant roles in the development of virus-mediated testicular injury and the generation of oxidative stress, as indicated by this finding (22). Furthermore, psychological stress is a powerful inducer of endogenous oxidative stress, and an abundance of evidence suggests that oxidative stress is associated with high-anxiety-related behavior, such as depression and post-traumatic-stress-disorder; however, the actual causal-effect association is yet to be known (157, 158). SARS-CoV-2, like SARS-CoV, is thought to employ a stress-evasion strategy that involves using amino acid sequences that mimic the adrenocorticotropic hormone (ACTH) of the host and, as a result, generate antibodies against these self ACTH molecules. This process subdues the stress response in the host, which is normally achieved by increasing cortisol levels, thereby reducing stress and inflammation in the organism (21, 159). As a result, uncontrolled inflammation continues to have a negative impact on the organs. The oxidative damage at the micro-level leads to membrane lipid peroxidation and sperm DNA fragmentation, both of which have a deleterious impact on testicular processes such as spermatogenesis and spermiogenesis. Male infertility is also associated with a decrease in sperm count and seminal volume, which can have a negative impact on reproductive outcomes and ultimately lead to infertility in males (160). Women infected with SARS-CoV are also more likely to have a disordered sexual function as a result of stress, which can have a poor influence on oocyte quality, menstrual cycle, and fertility (161).

## Mechanism of Virus-Mediated Male Reproductive Disruption

**Figure 1:**
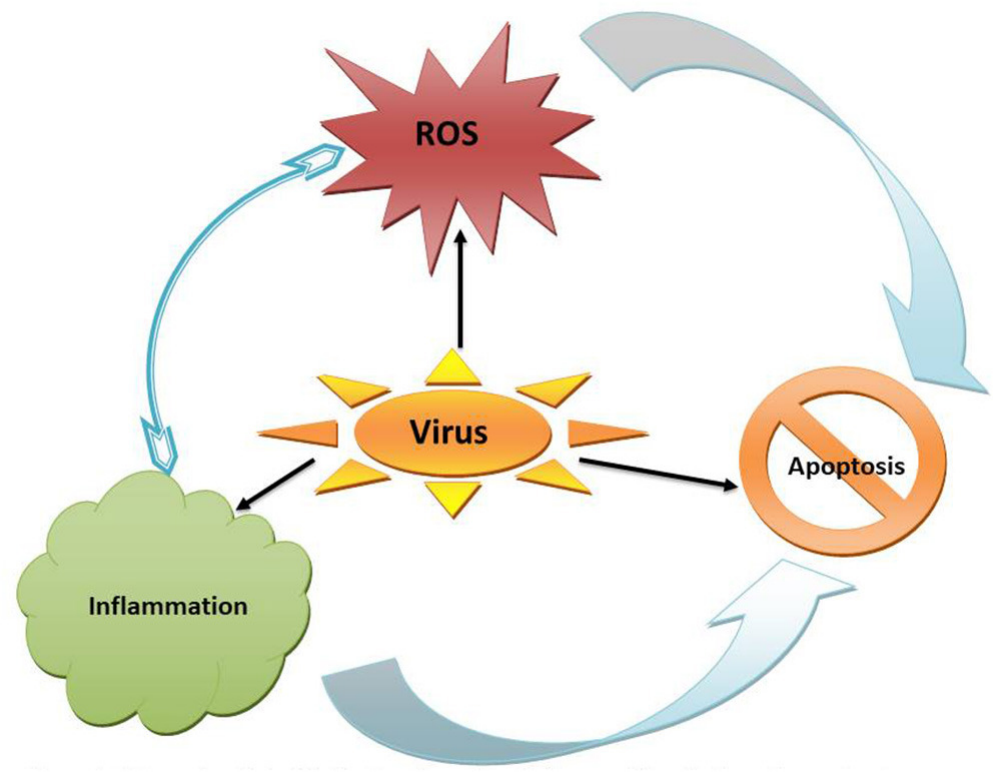
Enhanced generation of reactive oxygen species (ROS) could be a cause or consequence of inflammation; both of which can be triggered by viral infection and results in apoptosis.

**Figure 2:**
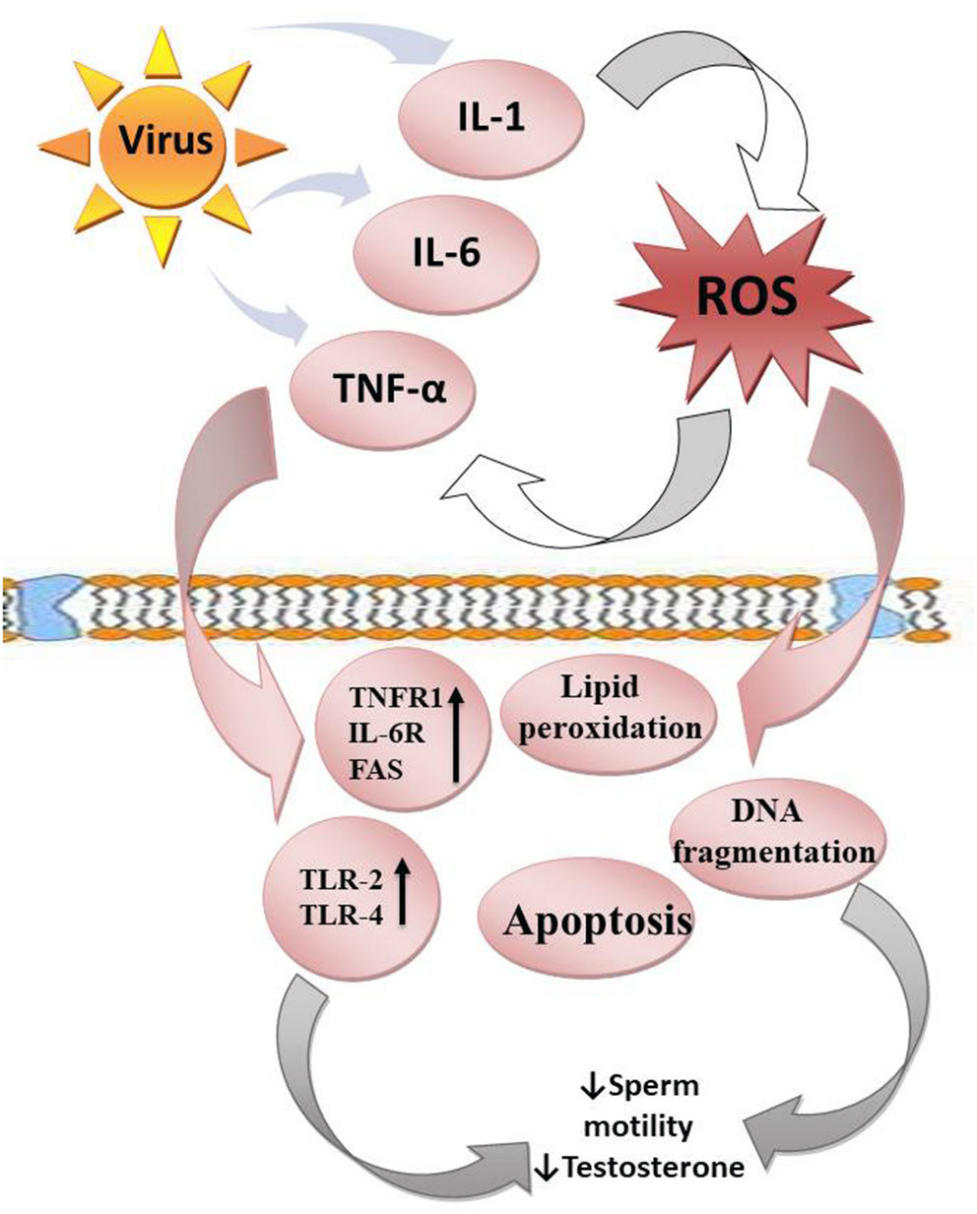
Viruses upregulate the release of proinflammatory cytokines (IL-1, IL-6, and TNF-α), which enhance ROS release. In response, ROS promote cytokine release. This leads to a vicious cycle. ROS attack the polyunsaturated fatty acids of the plasma membrane, resulting in lipid peroxidation. They also trigger DNA fragmentation and apoptosis of the testes and sperm cells. In addition, the cytokines upregulate TNFR1, IL-6R, FAS, TLR-2, and TLR-4, resulting in reduced sperm motility and steroidogenesis.

Virus-mediated male reproductive dysfunction has been associated with oxido-inflammatory damage (Table 1; Figures 1, 2). Viruses trigger inflammatory responses, resulting in urethritis (inflammation of the urethra), prostatitis (inflammation of the prostate), epididymitis (inflammation of the epididymis), and orchitis (inflammation of the testis). These viruses activate the innate immune system, which causes leukocyte infiltration and upregulation of proinflammatory cytokines (19, 20). Although this inflammatory response mediates the innate ability to combat viral infections (19), it may also induce ROS generation and redox imbalance with consequent oxidative damage (12, 164). These expose the reproductive organs, especially the urethra, prostate and epididymis, to inflammatory and oxidative damage. The epididymal spermatozoa and sperm cells on transit during ejaculation are also exposed to the harmful effects of the cytokines and ROS, leading to reduced sperm count and motility and reduced sperm cells with normal morphology.

**Table 1:** The impact and associated mechanism of viral infections.

**Virus**	**Family**	**Impact on male reproduction**	**Associated mechanism**
Influenza virus	Orthomyxoviridae	Alteration of conventional sperm parameters, sperm DNA damage (24).	Oxidative stress-mediated apoptosis (28).
Zika virus	Flaviviridae	Germ cell loss, impairment of Sertoli cell function (43), Alteration of conventional sperm parameters, androgen suppression (44)	Hyper-inflammatory response (11).
Mumps virus	Paramyxoviridae	Testicular atrophy, impaired steroidogenesis and spermatogenesis. Altered sperm quality (48). TNF-α-mediated disruption of the blood-testes-barrier (53).	Inflammation of the Leydig cells, Sertoli cells and germ cells (51), oxidative damage (51).
Human Immunodeficiency Virus (HIV)	Lentivirus	Hypogonadism and leukocytospermia (65), sertoli-cell-only syndrome, germ cell damage peritubular fibrosis, and impaired steroidogenesis and spermatogenesis (162), altered sperm quality (68).	Cytokine-driven oxidative stress (69).
Herpes Simplex Virus (HSV)	Herpesviridae	Low sperm count and reduced sperm motility (89, 90)	Inflammatory response (90).
Human Papillomavirus	Papillomaviridae	Alteration in conventional semen parameter (96).	Reduction of MHC I and disruption of interferon route (163).
Hepatitis viruses	HBV (Hepadnaviridae), HCV (Flaviviridae)	Alteration of conventional semen parameters (119), reduced circulatory testosterone and inhibin B (121)	Oxidative stress (124), Apoptosis (116).
Human parvovirus	Parvoviridae	Disruption of sperm cell development (132).	Germline transduction (132)
Ebola virus	Filoviridae	Disruption of blood-testis barrier (141), impaired steroidogenesis, low libido, erectile dysfunction (142, 143).	Inflammation-induced oxidative stress (144).
Coronavirus	Coronaviridae	Reduced testosterone level (146).	Activation of MAPK/ERK signaling-induced hyperinflammatory response (150).

The impact of these cytokines (interleukin-1, IL-1, interleukin-6, IL-6, and tumor necrosis factor-α, TNF-α) on the testes depends on their environmental influences (20). At physiological levels, they may play homeostatic roles in the gap junctional communication of the blood-testis barrier (165) and enhance the integrity of the immune-privileged testis, thus maintain optimal spermatogenesis (166). However, when there is an upregulation of these proinflammatory cytokines, they mediate blood-testis barrier restructuring *via* deregulation of Cldn 11 in the Sertoli cells (167). Proinflammatory cytokines may also activate TNFR1, IL-6R, and FAS expressed in the germ cells, resulting in apoptosis of the germ cells (168). In addition, cytokines mediate male infertility by impairing sperm motility and repressing testosterone synthesis through the activation of Toll-like receptors-2 and 4 (TLR-2 and TLR-4) located on the human spermatozoa membrane (52, 169).

Furthermore, proinflammatory cytokines activate ROS-dependent oxidative stress, which could in turn further exaggerate inflammatory response, resulting in vicious cycle of oxido-inflammatory response (170). The high polyunsaturated fatty acids (PUFA) content of the plasma membranes of the testis and spermatozoa makes them readily prone to ROS-driven oxidation, resulting in lipid peroxidation (171), loss of testicular and sperm cell membrane integrity, and raised non-specific permeability to ions (172, 173). Following the destruction of the testicular and sperm cell membrane, the nuclear chromatin is exposed to oxidative damage, leading to base modification, DNA fragmentation, and testicular and sperm cell apoptosis (74, 174–176). Hence, viral infections could cause direct modification of semen quality, damage to testicular and sperm DNA integrity, and production of anti-sperm antibodies, which may alter steroidogenesis, sperm motility and sperm-oocyte binding (99, 120, 123, 177, 178), thus reducing fertilization capacity *via* an inflammation-driven oxidative stress-mediated pathway.

Fascinatingly, antiviral drugs, which have even been proven effective against viral infection, have been shown to impair male fertility. Ribavirin has been demonstrated to reversibly damage germ cells *via* impairment of cell growth through inhibition of inosine monophosphate dehydrogenase, which is necessary for DNA and RNA guanosine triphosphate biosynthesis (118, 179), with consequent apoptosis (121, 180). In addition, interferon α-2b, which is effective against mumps infection, has been reported to cause oligoasthenozoospermia (181, 182). Steroids have also been shown to suppress testosterone production (183). Efavirenz, lamivudine, stavudine, nevirapine, and tenofovir, which are effective against most viral infections, including HIV, have also been reported to impair testicular and sperm integrity and function. Antiviral drugs have been reported to induce atrophy of the seminiferous tubules (72, 184) and resultant distortion of spermatogenesis (74, 164). Studies have also revealed that antiviral drugs led to reduced testosterone levels consequent to reduced Leydig cell mass (72, 74). Antiviral therapy did not just distort steroidogenesis and spermatogenesis; it culminates in testicular and sperm cell DNA fragmentation (74, 185, 186), erectile dysfunction and reduced fertility indices (74, 164).

## Management of Virus-Mediated Male Reproductive Disruption

In spite of the effectiveness of antiviral and antiretroviral drugs, and the improvement in the quality of life and life expectancy in people with viral infections, either acute or chronic, following antiviral therapy, the tendency of these drugs to impair male fertility remains a great concern. Assisted Reproductive Techniques (ART) using “sperm/semen washing” (SW) to reduce the seminal viral load provides a safety measure for serodiscordant men, especially those in their reproductive years and who wish to have children, with minimal risk of sexual and vertical transmission. Although Zamora et al. (187) reported a prevalence of 1.86% of retroviral positive semen after semen washing and recommended a post-wash retroviral screening on the semen before intracytoplasmic sperm injection (ICSI), semen washing and ICSI remains the current technique to reduce viral load. Also, it has been documented that currently, no semen processing techniques can eliminate seminal virus, especially ZIKV; the specific technique (intrauterine insemination, IUI, *in vitro* fertilization, IVF, or ICSI) used for medically assisted reproduction should be dependent on the cause of infertility (188).

Despite the available techniques in reducing viral load and preventing sexual and vertical transmission, two major challenges exist. First, achievement of sexual satisfaction. In addition, data on semen quality and sperm integrity in patients with viral infections after semen washing are scarce. Before semen washing, the viral infection might have induced sperm DNA fragmentation and apoptosis. Are these damages reversible even after sperm washing? Since semen washing and ART seems to be the current modality of achieving conception, especially in chronic viral infections, copulation may be a problem. However, safe the barrier method could be, scrotal transmission could pose another issue as in the case of HPV. Antioxidant therapy may be beneficial since oxidative stress has been implicated as a key player in viral infection-induced male infertility/subfertility. Although some studies have questioned the benefits of antioxidants in male infertility, inarguable pieces of evidence show that antioxidants alleviate oxidative stress-induced testicular and sperm damage. Vitamins (such as Vitamin C and E), carnitine, cysteine, and carotenoids have been shown to scavenge free radicals and act as energy sources (189–193). In addition to the free radical scavenging-ability of Coenzyme Q10 (CoQ10), it also serves as an intermediate in the mitochondrial transport (194), thus providing required energy. Folic acid preserves sperm DNA integrity (195), while selenium maintains redox balance and aids sperm motility (196). Zinc, on the other hand, maintains sperm chromatin stability (197). Furthermore, phytonutrients (such as flavonoids, polyphenols, and catechin compounds) scavenge free radicals and chelate metal ions, thus preserving sperm quality (198, 199). Arafa et al. (200) also demonstrated that *in vivo* antioxidant therapy (containing vitamins A, B6, B12, C, D3, E, K, thiamin, riboflavin, niacin, folate, biotin, pantothenic acid, iodine, zinc, selenium, copper, manganese, chromium, molybdenum, carnitine, arginine, CoQ10, cysteine, grapeseed extract, lycopene, and benfotiamine) for 3 months significantly improved conventional semen parameters, seminal oxidation-reduction potential and sperm DNA fragmentation. More so, Talevi et al. (201) showed that a combination of zinc, d-aspartate and CoQ10 *in vitro* treatment on human sperm enhanced sperm motility and reduced lipid peroxidation and sperm DNA fragmentation. The protective roles of antioxidant therapy on semen samples were also documented in a systemic review by Agarwal et al. (202). Hence, it is safe to state that using antioxidants as adjuvant therapy with appropriate antiviral/retroviral drugs may preserve testicular integrity and ensure optimal steroidogenesis and spermatogenesis, thus enhancing semen quality and sperm integrity and function. Also, treating semen samples with antioxidants before the appropriate medically assisted reproduction may enhance semen quality and sperm integrity and function.

## Conclusion and Future Perspective

This study reviews the possible mechanisms associated with the viral threats to male fertility. It elucidates the role of inflammation-led oxidative stress in the pathogenesis of viral infection-induced male infertility. Though semen washing and ART is the available modality of achieving fertility and preventing vertical and horizontal transmission, especially in chronic cases of infection, the use of antioxidants as adjuvant therapy is proposed. However, it is recommended that more clinical studies should be conducted to demonstrate the protective roles of antioxidants used as adjuvant therapy in viral infections and in the *in vitro* treatment of semen samples for those utilizing semen washing and artificial reproductive techniques.

## Author Contributions

RA and PS: conceptualization and design. RA, PS, and GA: methodology, resources, and supervision and validation. RA: project administration. RA, SD, PS, GA, MH, and AA: writing—original draft and writing—review and editing. All authors contributed to the article and approved the submitted version.

## Conflict of Interest

The authors declare that the research was conducted in the absence of any commercial or financial relationships that could be construed as a potential conflict of interest.

## Publisher's Note

All claims expressed in this article are solely those of the authors and do not necessarily represent those of their affiliated organizations, or those of the publisher, the editors and the reviewers. Any product that may be evaluated in this article, or claim that may be made by its manufacturer, is not guaranteed or endorsed by the publisher.
